# Circulating dipeptidyl peptidase 3 on intensive care unit admission is a predictor of organ dysfunction and mortality

**DOI:** 10.1186/s40560-021-00561-9

**Published:** 2021-08-24

**Authors:** Attila Frigyesi, Maria Lengquist, Martin Spångfors, Martin Annborn, Tobias Cronberg, Niklas Nielsen, Helena Levin, Hans Friberg

**Affiliations:** 1 Department of Clinical Medicine, Anaesthesiology and Intensive Care, Lund University, Lund, SE-22185 Sweden; 2 Skåne University Hospital, Intensive and Perioperative Care, Lund, SE-22185 Sweden; 3 Kristianstad Central Hospital, Anaesthesia and Intensive Care, Kristianstad, SE-29185 Sweden; 4 Helsingborg Hospital, Anaesthesia and Intensive Care, Helsingborg, SE-25187 Sweden; 5 Skåne University Hospital, Department of Neurology, Lund, SE-22185 Sweden; 6 Skåne University Hospital, Research and Education, Lund, SE-22185 Sweden

**Keywords:** Circulating dipeptidyl peptidase 3, Biomarker, Intensive care, Sepsis, Cardiac arrest, Trauma, Mortality, Organ dysfunction

## Abstract

**Background:**

Our aim was to investigate the prognostic potential of circulating dipeptidyl peptidase 3 (cDPP3) to predict mortality and development of organ dysfunction in a mixed intensive care unit (ICU) population, and for this reason, we analysed prospectively collected admission blood samples from adult ICU patients at four Swedish hospitals. Blood samples were stored in a biobank for later batch analysis. The association of cDPP3 levels with 30-day mortality and Sequential Organ Failure Assessment (SOFA) scores on day two was investigated before and after adjustment for the simplified acute physiology score III (SAPS-3), using multivariable (ordinal) logistic regression. The predictive power of cDPP3 was assessed using the area under the receiver operating characteristic curve (AUROC).

**Results:**

Of 1978 included consecutive patients in 1 year (2016), 632 fulfilled the sepsis 3-criteria, 190 were admitted after cardiac arrest, and 157 because of trauma. Admission cDPP3 was independently (of SAPS-3) associated with 30-day mortality with odds ratios of 1.45 (95% confidence interval (CI) 1.28–1.64) in the entire ICU population, 1.30 (95% CI 1.08–1.57) in the sepsis subgroup and 2.28 (95% CI 1.50–3.62) in cardiac arrest. For trauma, there was no clear association. Circulating DPP3 alone was a moderate predictor of 30-day mortality with AUROCs of 0.68, 0.62, and 0.72 in the entire group, the sepsis subgroup, and the cardiac arrest subgroup, respectively. By adding cDPP3 to SAPS-3, AUROC improved for the entire group, the sepsis subgroup, and the cardiac arrest subgroup (p = 0.023).

**Conclusion:**

Circulating DPP3 on admission is a SAPS-3 independent prognostic factor of day-two organ dysfunction and 30-day mortality in a mixed ICU population and needs further evaluation.

**Supplementary Information:**

The online version contains supplementary material available at (10.1186/s40560-021-00561-9).

## Introduction

A variety of patients with different severity of critical illness are treated in intensive care units (ICUs). The severity of illness on ICU admission is commonly assessed using the Simplified Acute Physiology Score III (SAPS-3), which includes laboratory and physiological measures and comorbidities. There is a need for novel prognostic and discriminatory biomarkers that could improve and simplify the complex SAPS-3 model or replace a standalone biomarker as lactate.

Circulating dipeptidyl peptidase 3 (cDPP3), also known as red cell angiotensinase, is an enzyme with a molecular mass of 83 kDa [1]. The cDPP3 gene is ubiquitously expressed, but its exact function is not understood. Recent findings indicate a role not only in protein metabolism but also in blood pressure regulation, pain modulation, and inflammatory processes based on the substrate specificity of cDPP3 [2]. The main features of the peptidase come from its ability to cleave different substances, e.g. enkephalins, endorphins, and specifically, angiotensin II [3–5], but the role of extracellular cDPP3 in pathology and disease is largely unknown [6–8].

Our aim was to investigate the prognostic role of cDPP3 levels on ICU admission in relation to 30-day mortality and the day-two SAPS-3 score. Furthermore, we wanted to examine the role of cDPP3 in three well-characterised and defined subgroups in the ICU: sepsis, cardiac arrest, and trauma. Our primary outcome was 30-day mortality, and the secondary outcome was organ dysfunction on day two.

## Materials and methods

### Study design and setting

This study is a multicentre observational study where blood samples were prospectively collected and retrospectively analysed. The Standards for Reporting of Diagnostic Accuracy Studies (STARD) guidelines were followed [9].

### Participants

All patients admitted to any of four mixed surgical and medical ICUs in southern Sweden in 1 year (2016) were evaluated for eligibility. Adult patients with valid blood samples were included. The sepsis subgroup was identified using the sepsis-3 criteria, a detailed description of which has been presented in a previous article [10]. The cardiac arrest subgroup was identified using the International Statistical Classification of Diseases and Related Health Problems (ICD) code I46.9 at ICU discharge. The trauma subgroup was identified through multi-trauma as the reason for ICU admission. When transfers occurred between the participating ICUs, follow-up data were merged to form cohesive ICU admissions.

### Variables

The third version of the Simplified Acute Physiology Score (SAPS-3) was calculated [11, 12] based on physiological parameters and laboratory findings recorded within 1 h before/after ICU admission.

The Sequential Organ Failure Assessment (SOFA) score was recorded daily during the ICU stay [13]. The day-two SOFA was the first score to be based on a full 24-h period following admission and therefore used. The total SOFA score was based on the available SOFA subscores.

### Data sources

Background and survival data were extracted from the Patient Administrative System for Intensive Care Units (PASIVA). PASIVA is the portal by which the treating physician and nursing staff submit prospectively collected laboratory and physiological data to the Swedish Intensive Care Registry (SIR). PASIVA is synchronised with the Swedish population register, which contains survival data.

The blood samples were collected in EDTA (ethylenediaminetetraacetic acid) vacutainers at ICU admission, centrifuged, aliquoted, frozen, and stored in the SWECRIT biobank at Region Skåne (registration no. BD-47). For inclusion, the sample had to be collected within 6 h after ICU admission. If the sampling time was missing, samples were included if the time of freezing was within the 6-h time frame. Frozen plasma samples were shipped and batch analysed in a blinded fashion on thawed samples in 2019 at the laboratory of SphingoTec GmbH (Hennigsdorf, Germany).

A median of 14.5 ng/mL and a 97.5 percentile of 40.0 ng/mL was previously identified in a group of healthy volunteers aged 56–87 years^1^ (personal communication SphingoTec GmbH).

### Bias

Potential selection bias was investigated in a comparison of baseline characteristics between included and excluded patients.

### Study population size

The study used a convenience sample, and the study size was based on the number of ICU admissions and corresponding valid blood samples during the study period.

### Quantitative variables

The distribution of cDPP3 levels was highly skewed, and therefore all analyses were carried out on a logarithmic scale (base 10).

### Statistical methods

The association between cDPP3 levels and 30-day mortality was analysed using multivariable logistic regression, also including the SAPS-3 score as an independent variable. Circulating DPP3 was included as a z-normalised (subtracted mean and then divided by one standard deviation) base-10 log-transformed independent variable. The regression models were evaluated with the Hosmer-Lemeshow goodness-of-fit test with ten groups, and only models resulting in non-significant tests were reported [14]. The association between cDPP3 levels and day-two SOFA scores were analysed using multivariable ordinal logistic regression [15].

To evaluate the additional value of cDPP3 to SAPS-3 in the logistic regression model, we calculated the area under the receiver operating characteristic curve (AUROC) [16]. Differences in AUROCs were tested with the method of DeLong et al. [17].

Modified correlation networks for cDPP3 and day-two SOFA scores were visualised using the corrr^2^ package in R.The product of the Kendall *τ* and the 1−*p* were visualised so that highly correlated points (SOFA subscores or cDPP3) with low *p*-values were close and had strong vertices. The strength of the connections was indicated by the colour intensity of the vertices. The proximity of points was based on multidimensional clustering.

### Ethics and consent

Ethical approval was obtained from the Regional Ethical Review Board of Lund, Sweden (registration nos. 2015/267 and 2017/802). Patients and their next-of-kin were given the possibility to opt out.

## Results

### Participants

Out of 2546 adult ICU admissions, 1978 (78%) had valid blood samples at ICU admission and did not opt out. Of the 1978 admissions, the sepsis subgroup constituted 32% (n = 632), the cardiac arrest subgroup 9.6% (n = 190) and the trauma subgroup 7.9% (n = 157). There was no overlap between the sepsis and the cardiac arrest subgroups (cardiac arrest was an exclusion criterion for sepsis). Of the trauma group 6.5% were identified as having sepsis 3 on admission, and 1.3% of the trauma patients also had a cardiac arrest. See Supplementary 1 F1 for a flow chart.

The median time from ICU admission to blood sampling for cDPP3 was 21 min (interquartile range (IQR) 15–40 min). The mean time from ICU admission to the start of day-two SOFA was 17.5 h (standard deviation 6.2 h).

### Descriptive data

**Fig. 1:**
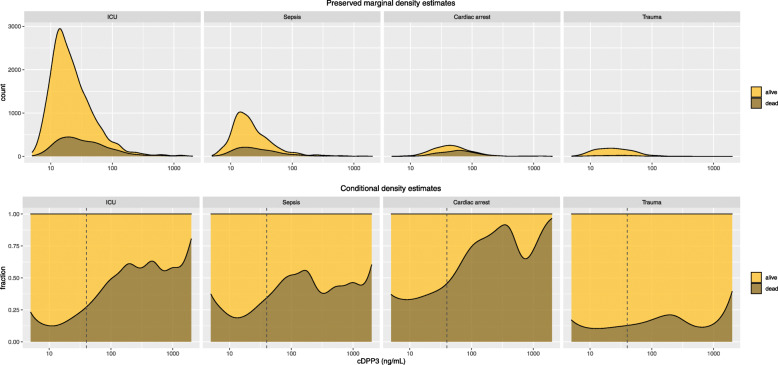
Distribution of circulating dipeptidyl peptidase 3 (cDPP3) level in the subgroups sepsis, cardiac arrest and trauma stratified on alive or dead at 30 days. The top row shows the distribution (with preserved marginal densities) whereas the bottom row shows the fraction (conditional density estimate) of dead at 30 days for different cDPP3 levels. The vertical lines at cDPP3 40 ng/mL indicate the upper normal limit

The median (IQR) cDPP3 concentration was 19 (13–33) ng/mL for the entire ICU population, 19 (13–31) ng/mL for the sepsis group, 44 (29–69) ng/ml in the cardiac arrest group and 22 (14–38) in the trauma group, see Table 1. See Fig. 1 for the distribution of cDPP3.

**Table 1:** Descriptive statistics for the whole ICU population, the sepsis-3 subgroup, the cardiac arrest subgroup and the trauma subgroup. If not stated otherwise, values represent medians (interquartile ranges, IQR)

	ICU	Sepsis	Cardiac arrest	Trauma
Number of patients	1978	632 (32%)	190 (9.6%)	157 (7.9%)
Women (%)	39	40	27	23
ICU length-of-stay (days)	1.7 (0.8–3.8)	2.5 (1.1–5.5)	2.4 (0.97–4.3)	1.8 (0.93–4.1)
ICU mortality (%)	11	14	34	4.5
30-day mortality (%)	22	28	54	12
SAPS-3 score	59 (47–71)	66 (57–77)	76 (66–87)	48 (39–59)
Day-one SOFA score	7 (4–10)	8 (6–11)	10 (8–13)	5 (2–7)
Day-two SOFA score (n = 995)	8 (5–10)	8 (6–11)	10 (8–12)	6 (4–9)
CRRT during ICU stay (%)	9.2	15	7.4	0.6
**Box I**				
Age (years)	66 (54–74)	69 (61–76)	68 (60–76)	55 (33–70)
Comorbidities				
- Cancer therapy (%)	4.6	6.3	4.2	0.6
- Chronic heart failure (%)	5.5	7.0	10	1.3
- Haematological cancer (%)	2.6	5.2	1.0	0
- Liver cirrhosis (%)	1.1	1.6	0	0
- Cancer (%)	10.4	10.4	7.9	1.3
Vasoactive drugs before ICU (%)	44	47	64	21
**Box II**				
Surgical status at ICU admission				
- No surgery (%)	74	85	94	83
**Box III**				
GCS	13 (10–15)	13 (10–15)	3 (3–8)	10 (6–15)
Total bilirubin (*μ*mol/L)	9 (6–15)	10 (7–19)	8 (5–12)	8 (5–12)
Max. temperature (^∘^C)	37.0 (36.3–37.6)	37.3 (36.5–38.1)	36.0 (35.5–36.8)	37.0 (36.0–37.5)
Max. creatinine (*μ*mol/L)	93 (70–145)	119 (79–205)	112 (87–149)	87 (74–111)
Max. heart rate (bpm)	100 (80–118)	107 (90–122)	98 (80–115)	90 (80–110)
Max. leukocyte count (×10^9^/L)	13 (8.8–18)	13 (8.4–19)	16 (12–21)	14 (11–19)
Min. pH	7.34 (7.24–7.41)	7.32 (7.20–7.40)	7.19 (7.04–7.3)	7.34 (7.29–7.40)
Min. platelet count (×10^9^/L)	221 (161–286)	218 (147–304)	222 (165–270)	212 (171–261)
Min. systolic blood pressure (mmHg)	100 (80–120)	91 (75–115)	85 (68–109)	104 (90–130)
Oxygenation				
- Respiratory support (%)	58	59	86	53
- FiO_2_ (%)	50 (40–70)	60 (40–80)	60 (50–80)	45 (40–57)
- PaO_2_ (kPa)	13 (9.8–18)	11 (9.0–15)	13 (11–22)	16 (11–24)
**Other**				
cDPP3 (ng/mL)	19 (13–33)	19 (13–31)	44 (28–69)	22 (14–38)

Box I-III refers to the subsections of the SAPS-3 scoring system. *ICU* intensive care unit, *SAPS-3* simplified acute physiology score III, *SOFA* Sequential Organ Failure Assessment, *CRRT* continuous renal replacement therapy, *GCS* Glasgow coma scale, *FiO*_2_ fraction of inspired oxygen, *PaO*_2_ arterial partial pressure of oxygen, *cDPP3* circulating dipeptidyl peptidase 3

The female to male ratio, ICU length-of-stay (LOS), ICU mortality, 30-day mortality, morbidity as measured by SAPS-3, median age, and factors included in the SAPS-3 score are presented in Table 1.

The patients excluded from the study were slightly younger, had lower mortality, and a shorter ICU length-of-stay (Supplementary 1 T1). The excluded group included fewer sepsis patients but more trauma patients. The fraction of missing parameters was mostly low (Supplementary 1 T2).

### Unadjusted outcomes

The association between cDPP3 levels and mortality is shown in Fig. 1. The unadjusted associations between cDPP3 levels and 30-day mortality and day-two SOFA scores, respectively, from (ordinal) logistic regressions, are reported as odds ratios (ORs) in Table 2. The logistic regression models resulted in an AUROC of 0.68 (95% CI 0.65–0.71) for the entire population, 0.62 (95% CI 0.57–0.67) for the sepsis group, 0.72 (95% CI 0.65–0.80) for the cardiac arrest group, and 0.53 (95% CI 0.40–0.67) for the trauma group.

**Table 2:** Odds ratios (ORs) for dipeptidyl peptidase 3 (DPP3) from univariate (ordinal) logistic regressions on 30-day mortality and day-two SOFA scores

Univariate	ICU			Sepsis			Cardiac arrest			Trauma		
Outcome	OR	95% CI	*p*-value	OR	95% CI	*p*-value	OR	95% CI	*p*-value	OR	95% CI	*p*-value
Mortality	**1.88**	1.69–2.10	<0.001	**1.49**	1.26–1.78	<0.001	**2.57**	1.74–3.97	<0.001	1.12	0.63–1.94	0.69
Cardiovasc. SOFA	**1.28**	1.14–1.43	<0.001	1.03	0.86–1.24	0.75	**1.69**	1.09–2.72	0.025	1.24	0.82–1.89	0.31
Resp. SOFA	**1.29**	1.15–1.45	<0.001	1.18	0.98–1.44	0.088	1.10	0.72–1.71	0.65	1.03	0.68–1.56	0.88
Renal SOFA	**1.52**	1.35–1.72	<0.001	**1.45**	1.20–1.76	<0.001	**1.72**	1.11–2.67	0.014	1.33	0.81–2.21	0.25
Hepatic SOFA	**1.69**	1.46–1.95	<0.001	**1.85**	1.48–2.32	<0.001	1.84	0.98–3.45	0.054	1.89	1.01–3.70	0.052
Neurol. SOFA	**1.62**	1.44–1.82	<0.001	**1.32**	1.10–1.59	0.0032	**3.28**	1.81–6.29	0.035	1.36	0.85–2.18	0.20
Coag. SOFA	**1.46**	1.29–1.65	<0.001	**1.57**	1.28–1.94	<0.0012	**1.73**	1.11–2.72	0.016	**1.57**	1.01–2.51	0.050

*CI* confidence interval, *SOFA* Sequential Organ Failure Assessment

### SAPS-3 adjusted mortality

The multivariable logistic 30-day mortality regression ORs for cDPP3 after correction for SAPS-3 were 1.45 (95% CI 1.28–1.64, *p*<0.001) in the entire ICU population, 1.30 (95% CI 1.08–1.57, *p*=0.0050) in the sepsis subgroup, 2.28 (95% CI 1.50–3.62, *p*<0.001) for the cardiac arrest subgroup, and 1.11 (95% CI 0.58–2.10, *p*=0.74) in the trauma subgroup.

In the entire population, the logistic regression models using SAPS-3 alone resulted in an AUROC of 0.81 (95% CI 0.79–0.83) versus 0.82 (95% CI 0.80–0.84) when cDPP3 was added (p = 0.0049). For the sepsis population, the AUROC was 0.73 (95% CI 0.69–0.77) using SAPS-3 alone versus 0.74 (95% CI 0.70–0.78) adding cDPP3 (p=0.023). For the cardiac arrest population, the AUROC was 0.74 (95% CI 0.67–0.82) using SAPS-3 alone versus 0.80 (95% CI 0.73–0.86) when cDPP3 was added (p = 0.0091). For the trauma population, the AUROC was 0.84 (95% CI 0.77–0.92) using SAPS-3 alone versus 0.84 (95% CI 0.77–0.92) when cDPP3 was added (p = 0.59).

### Correlation analyses

**Fig. 2:**
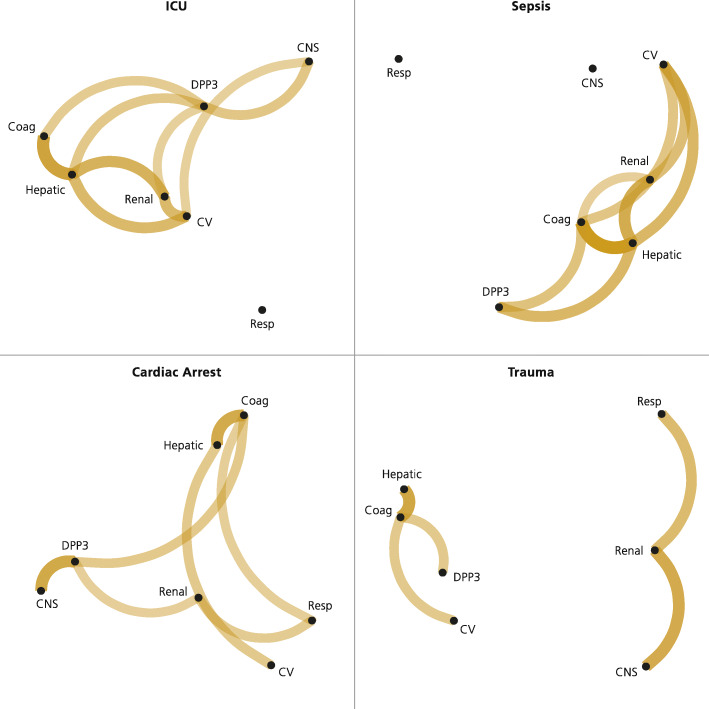
Modified correlation networks for circulating dipeptidyl peptidase 3 (cDPP3) and day-two SOFA scores visualised using the corrr (https://corrr.tidymodels.org) package in R. The product of the Kendall *τ* and 1−*p* were visualised so that highly correlated points with low *p*-values are close. The proximity of the points is based on multidimensional clustering. The association is also shown by the colour intensity of the vertices (so that unconnected points have negligible association). SOFA, Sequential Organ Failure Assessment; ICU, intensive care unit; CV, cardiovascular

Modified correlation networks (to visualise the strength of correlation) between cDPP3 and day-two SOFA scores are visualised (as described in the Statistics section) in Fig. 2. It was evident that the day-two hepatic SOFA scores were (significantly) correlated with the day-two coagulation SOFA scores. In the whole population, cDPP3 was positively correlated with the neurological SOFA score (CNS), the hepatic/coagulation group, and the renal/cardiovascular group. In sepsis, cDPP3 was positively (and significantly) correlated with the hepatic/coagulation group. In cardiac arrest, the positive correlation (and significance) for cDPP3 was strongest for neurological SOFA.

## Discussion

In the present study, we investigated the prognostic capability of cDPP3 in a large, general ICU population and its association with mortality and organ dysfunction. Our ICU population had three well-defined subgroups: sepsis, cardiac arrest, and trauma, which comprised half of the study population. The remaining patients were a mix of medical, surgical, and postoperative patients, normally cared for in a general, mixed ICU.

We found that the cDPP3 level on admission was a SAPS-3 independent prognostic factor for 30-day mortality. The added (to SAPS-3) prognostic capability of cDPP3 to predict mortality was assessed using AUROC and was most pronounced in the cardiac arrest subgroup. We also found that the cDPP3 level on admission was associated with all types of day-two organ dysfunction as defined by SOFA.

Circulating DPP3 cleaves angiotensin II and can thus be surmised to have a role in cardiovascular compromise as Dépret demonstrated in burns patients [18]. Deniau et al. investigated the association of cDPP3 levels and mortality in cardiogenic shock patients and the association between high cDPP3 and organ function in a heart failure model in mice [7]. They demonstrated that cDPP3 is a myocardial depressant factor associated with mortality in severe heart failure patients. Furthermore, inhibition of cDPP3 by Procizumab, a specific cDPP3 antibody, improved hemodynamics in a mouse model of heart failure. Takagi et al. showed that cDPP3 values were higher in refractory cardiogenic shock after acute myocardial infarction [8].

Circulating DPP3 is not cleared by the kidneys. Angiotensin II is not the only substrate for DPP3; there are also metenkephalin and leucine enkephalin. Enkephalins are endogenous opioids that are widely expressed and act primarily on *δ*-opioid receptors, which are primarily expressed in the central nervous system and the kidney. It has been suggested that enkephalins have a regulatory role in kidney function, such as inducing diuresis and natriuresis [19]. Although elevated cDPP3 was associated with all types of organ dysfunction, the correlation network analysis (Fig. 2) revealed that elevated cDPP3 was mainly associated with day-two renal, neurologic, hepatic and coagulation dysfunction.

Rehfeld and colleagues found cDPP3 to be elevated in sepsis patients and that higher levels were associated with the severity of disease [6]. We have shown here that cDPP3 was a prognostic factor for organ dysfunction on day two and a SAPS-3 independent predictor of 30-day mortality in sepsis. The correlation network analysis revealed that in sepsis, cDPP3 was primarily associated with hepatic and coagulation dysfunction and, to a somewhat lesser degree, renal dysfunction and neurological dysfunction.

In trauma, we could not show that cDPP3 improved mortality prediction as compared to SAPS-3 (possibly due to lacking power). Elevated cDPP3 was only associated with coagulation dysfunction (and borderline significant with hepatic dysfunction), which was confirmed by the correlation network analysis.

The strong association with day-two neurological SOFA in the cardiac arrest subgroup, which was also confirmed in the correlation network analysis, is suggestive of cDPP3 being a marker of brain injury. It is also worth noting how sharply mortality increases as cDPP3 exceeds the upper normal level of 40 ng/mL. Compared to the entire ICU population, the cardiac arrest subgroup was more severely ill on admission, reflected in higher SAPS-3 and SOFA scores. For cardiac arrest patients, the day-two neurological SOFA is typically determined by the level of consciousness on admission since a vast majority of cardiac arrest patients will still be sedated as part of a target temperature management (TTM) strategy [20].

Since we did not measure DPP3 in the cerebrospinal fluid in this study, we refrain from making direct assumptions about DPP3 activity on central nervous system activity. In this study, we report an association between high DPP3 blood levels at admission and increased neurological SOFA score, an association that deserves further exploration, including serial samples during ICU care. Measurement of DPP3 activity in the cerebrospinal fluid would also be of great interest. Enkephalin, an important signalling molecule in the brain, is a well-known DPP3 substrate, and the correlation between DPP3 concentrations in the bloodstream and the central nervous system should be investigated in future studies.

To summarise the association of cDPP3 and organ dysfunction, our findings suggest that there may be a hepatic-coagulation dysfunction component in cDPP3 elevation in the entire population and in the subgroups sepsis, cardiac arrest and trauma as well as there may be a brain injury component primarily in cardiac arrest.

A strength of our study is the well-defined subgroups. The sepsis subgroup was carefully characterised and described in a previous study [10]. The cardiac arrest and trauma subgroups are common diagnoses in the ICU and generally easily identified. Another strength is that we adjusted for age, comorbidities, and acute severity of illness through the validated SAPS-3 score. After adjustments, our results are still robust, showing that cDPP3 provides clinically important additional prognostic information in a general ICU population, not captured by SAPS-3.

### Limitations

A weakness of our study is that we are confined to admission samples. Another weakness is that only half of the ICU population was characterised into broad diagnostic subgroups.

It is, however, important to remember that the predictive value of cDPP3 is dependent on the type of model, and non-logistic interactions with SAPS-3 or any of its constituent variables may not be captured using logistic regression. Machine learning methods, such as neural networks, will likely improve the predictive power of DPP3 but requires even larger data sets [21].

### Interpretation

To our knowledge, this is the first study investigating the importance of cDPP3 levels on ICU admission, opening up a novel possible path of risk assessment of critically ill patients. Additionally, an improved understanding of the role of cDPP3 in the intensive care setting may offer therapeutic possibilities in the future [7].

### Generalisability

The multicentre approach, with ICUs from a large university hospital and regional hospitals, makes the results applicable to ICU populations in regions with similar demographics as Sweden.

## Conclusion

In this study, we found cDPP3 to be a SAPS-3 independent predictor of 30-day mortality and associated with subsequent organ dysfunction in a general ICU population, as well as in sepsis, cardiac arrest, and trauma.

## Supplementary Information

**Additional file 1** Supplementary figure 1 and 2, table 1 and 2.

## Data Availability

The data that support the findings of this study are available on request from the corresponding author. The data are not publicly available due to privacy.

## Abbreviations

cDPP3: circulating dipeptidyl peptidase 3
ICU: intensive care unit
SOFA: Sequential Organ Failure Assessment
SAPS-3: simplified acute physiology score III
EDTA: ethylene diamine tetraacetic acid-plasma
IQR: interquartile range
LOS: length-of-stay
OR: odds ratio

## Footnotes

1 https://www.malmo-kohorter.lu.se/mpp

2 https://corrr.tidymodels.org
