# Supplementary material for: Circulating dipeptidyl peptidase 3 on intensive care unit admission is a predictor of organ dysfunction and mortality
Source: J Intensive Care. 2021 Aug 24;9:52. doi: 10.1186/s40560-021-00561-9 (PMC8386069; doi:10.1186/s40560-021-00561-9)
Supplement: Supplementary file 1 — Additional file 1 Supplementary figure 1 and 2, table 1 and 2. [file 40560_2021_561_MOESM1_ESM.pdf]

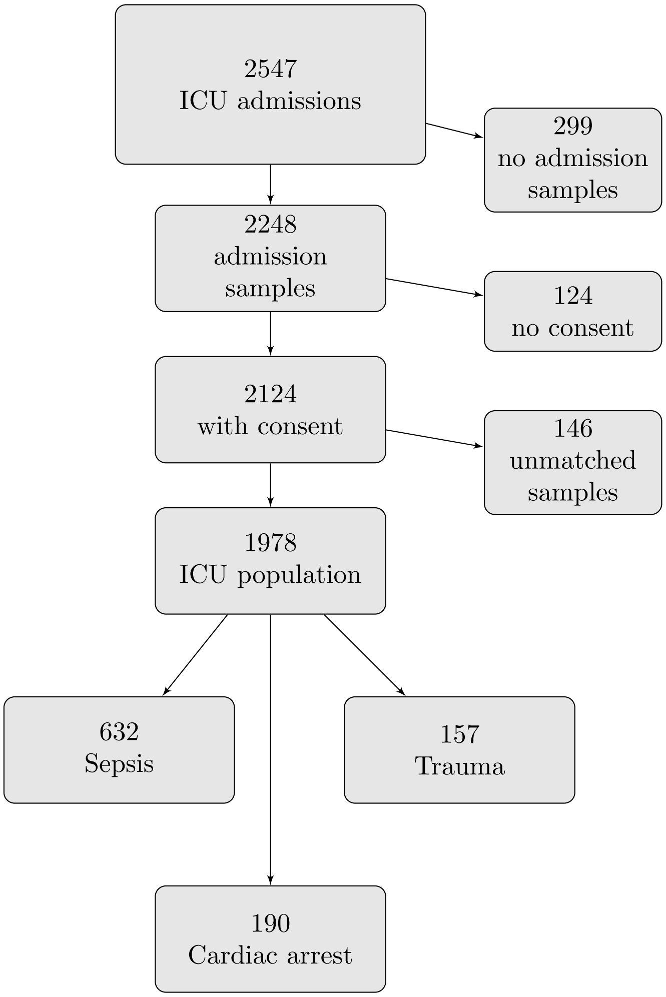

Figure 1 Flow chart of ICU admissions, admission samples and consent.

Table 1 Descriptive statistics for the included and the excluded groups.. If not stated otherwise, values represent medians (inter quartile ranges, IQR). Box I-III refers to the subsections of the SAPS-3 scoring system. *LOS: length of stay, ICU: intensive care unit, SAPS-3: simplified acute physiology score III, SOFA: Sequential Organ Failure Assessment, CRRT: continuous renal replacement therapy, GCS: Glasgow coma scale, FiO_2: fraction of inspired oxygen, PaO_2: arterial partial pressure of oxygen, cDPP3: circulating dipeptidyl peptidase 3.*

	included	excluded	p-value
Number of patients	1978	569	<0.001
Women (%)	39	37	0.39
Age (years)	66 (54-75)	62 (37-73)	<0.001
ICU LOS (days)	1.7 (0.8-3.8)	1.1 (0.5-2.8)	<0.001
ICU mortality (%)	11	13	0.29
30-day mortality (%)	22	24	0.19
SAPS-3 score	59 (47-71)	54 (42-67)	<0.001
Day-two SOFA score	8 (5-10)	7 (4-9)	0.0081
Sepsis (%)	36	23	<0.001
Cardiac arrest (%)	9.6	8.4	0.39
Trauma (%)	8.5	12	0.0086
No surgery (%)	74	75	0.81

Table 2 Missing data for the whole ICU population. Variables not reported did not have any missing data. Box III refers to the subsection of the SAPS-3 scoring system. Lactate was only reported for patients with sepsis. *SOFA: Sequential Organ Failure Assessment, GCS: Glasgow coma scale, FiO_2: fraction of inspired oxygen, PaO_2: arterial partial pressure of oxygen.*

	Missing (%)
Day-one SOFA score	16
Day-two total SOFA score	49
Day-two respiratory SOFA score	49
Day-two renal SOFA score	50
Day-two hepatic SOFA score	53
Day-two neurological SOFA score	49
Day-two coagulation SOFA score	51
**Box III**	
GCS	0
Total bilirubin	7
Max. temperature	1
Max. creatinine	5
Max. heart rate (bpm)	0.5
Max. leukocyte count	6
Min. pH	4
Min. platelet count	7
Min. systolic blood pressure	1
Oxygenation	
- Respiratory support	12
- FiO_2	12
- PaO_2 (kPa)	49
Lactate (% of sepsis subgroup)	1

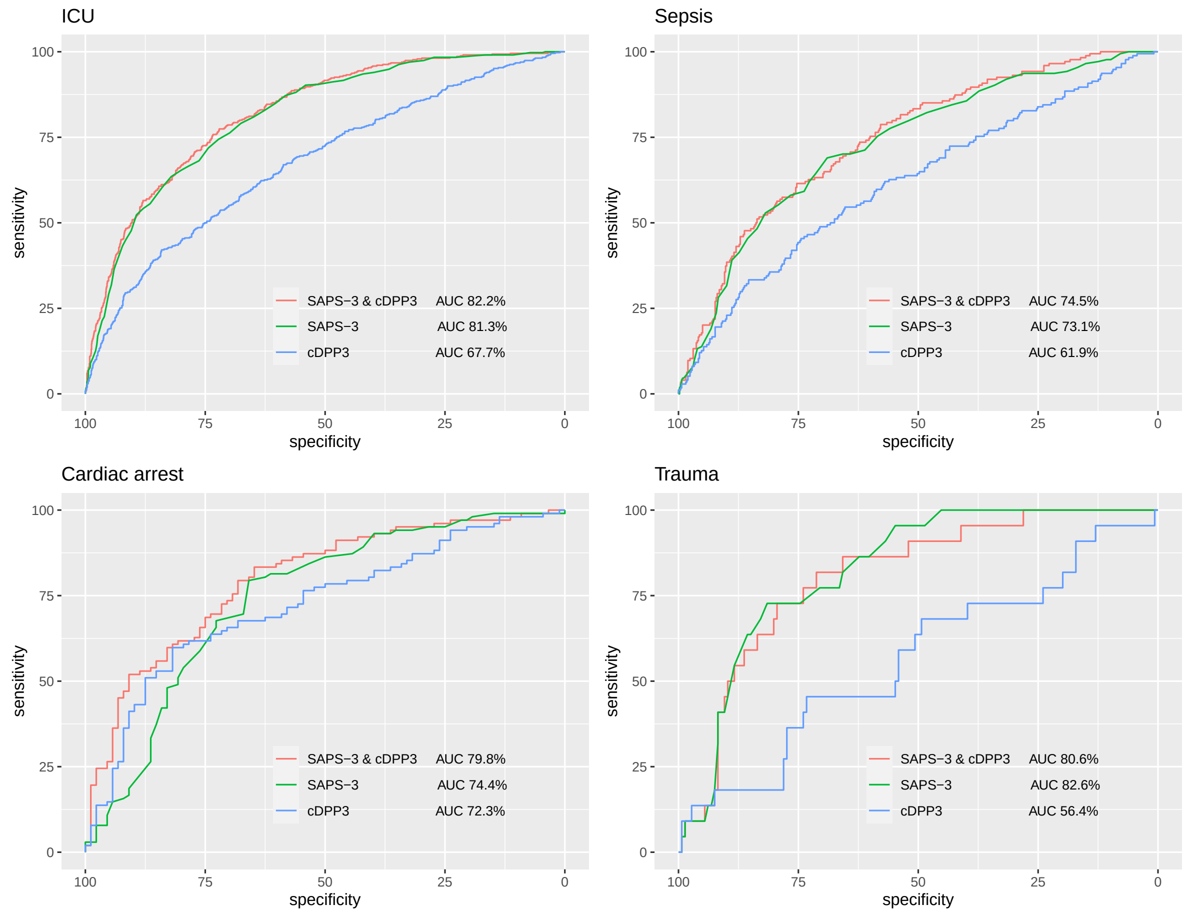

Figure 2 Receiver characteristic curves for three logistic regression models on 30-day mortality. The area under the curve (AUC) is equivalent to the c-statistic. *ICU, intensive care unit, SAPS-3, simplified acute physiology score III, cDPP3: circulating dipeptidyl peptidase, AUC: area under curve.*
